# Moving beyond the glial scar for spinal cord repair

**DOI:** 10.1038/s41467-019-11707-7

**Published:** 2019-08-28

**Authors:** Elizabeth J. Bradbury, Emily R. Burnside

**Affiliations:** King’s College London, Regeneration Group, The Wolfson Centre for Age-Related Diseases, Institute of Psychiatry, Psychology & Neuroscience (IoPPN), Guy’s Campus, London Bridge, London, SE1 1UL UK

**Keywords:** Glial biology, Regeneration and repair in the nervous system, Diseases of the nervous system

## Abstract

Traumatic spinal cord injury results in severe and irreversible loss of function. The injury triggers a complex cascade of inflammatory and pathological processes, culminating in formation of a scar. While traditionally referred to as a glial scar, the spinal injury scar in fact comprises multiple cellular and extracellular components. This multidimensional nature should be considered when aiming to understand the role of scarring in limiting tissue repair and recovery. In this Review we discuss recent advances in understanding the composition and phenotypic characteristics of the spinal injury scar, the oversimplification of defining the scar in binary terms as good or bad, and the development of therapeutic approaches to target scar components to enable improved functional outcome after spinal cord injury.

## Abstract (web summary)

The scar formation that occurs following spinal cord injury has properties that are distinct to scars seen in other areas of the CNS, and in other tissues. Here the authors discuss the components of the spinal cord injury scar and how it can have both detrimental and positive roles in relation to recovery.

## Introduction

It is estimated that more than 27 million people worldwide are living with long-term disability following a spinal cord injury^1^, 90% of which result from trauma and 10% as a secondary consequence of disease. Following traumatic spinal cord injury, death of spinal neurons at the injury level leads to paralysis of denervated musculature and the disruption of long spinal tracts leads to loss of sensation and motor control—injured descending axonal projections can no longer innervate motor neuron pools below the injury level, and injured ascending axonal projections can no longer provide appropriate transmission of sensory information to the brain. This results in the dysregulation of multiple organ systems throughout the body and a devastating loss of function^2^. Despite recent progress in developing experimental therapeutics aimed at enhancing tissue repair and neuroplasticity, there are still no effective pathology-modifying or regenerative treatments available to spinal injured individuals^3,4^. Following diagnosis and acute medical interventions to stabilize clinical status, the outcome is largely determined by the management of resultant symptoms, and rehabilitation to maximize residual neural function^2^.

The lack of repair following spinal cord injury is due to both cell intrinsic factors and the extrinsic injury environment. Neurons of the adult mammalian central nervous system (CNS) have low intrinsic regenerative ability due to a lack of growth driving signals, and suboptimal availability or arrangement of subcellular machinery to enable growth cone reformation and axonal elongation^5^. Experimental efforts to unlock regeneration potential at the level of the cell body of the neuron have focussed on growth signalling pathways, individual regeneration-associated genes, and transcriptional and epigenetic networks^6–9^. Regenerative strategies have also aimed to increase synthesis and transport of materials required for growth, and to modulate axonal cytoskeletal dynamics to promote elongation or branching^6,7,10–12^.

The injury microenvironment also plays a key role in limiting functional repair after spinal cord injury, an important component of which is the formation of a scar. As a healing response, the scar acts to spatially contain and isolate damage. However, reactive injury processes fail to restore spinal tissue architecture and composition, pathology continues to propagate and the tissue within and around the scar remains dysfunctional. Moreover, within the scar there are extracellular factors which themselves actively inhibit restoration of function. These act acutely and chronically to prohibit compensatory changes in neurons which, perhaps if overcome, could transform the scar into a more effective repair process which both isolates damage and generates an environment in which injury could be surmountable.

Here we review recent literature regarding the composition and role of the spinal injury scar, including processes leading to scar formation and maintenance, the cellular and extracellular components of the scar, and how these interact with other mediators of tissue pathology. We discuss the complexities of the scar and its seemingly opposing roles, often classified in an overly simplistic manner as good or bad, and finally we discuss the potential for therapeutic targeting of the scar to achieve functional repair of the injured spinal cord.

## Scar formation and maintenance

### Tissue scarring in the CNS

Injury to any tissue results in a healing response, the purpose of which is firstly to curtail damage and restore homoeostasis and then, where possible, to restore tissue and organ function. Inflammation, tissue reformation (involving cell proliferation and/or migration) and tissue remodelling are conserved repair processes, although their success in restoring function varies across different tissues. A scar consists of the cells and extracellular matrix (ECM) formed as the result of attempted wound repair. In many organs, the formation of a scar is associated with a resolution phase and restoration of key functions of the tissue. The healed tissue may not directly recapitulate the pre-injury state but it regains some ability to execute its original function^13^. However, the process of tissue scarring in the CNS is more complex than for many other tissues, and is associated with chronic non-resolving pathology.

Disease and injury to the CNS is almost always accompanied by some degree of reactive gliosis, inflammation and scarring. Scar tissue and associated deposition of ECM molecules such as chondroitin sulfate proteoglycans (CSPGs, discussed in more detail below) has been reported in humans and experimental animal models following traumatic brain injury^14^ and stroke^15^, as well in neurodegenerative disorders such as Alzheimer’s Disease^16^, and disorders with a predominantly demyelinating and inflammatory pathology such as multiple sclerosis^17,18^. However, despite the occurrence of reactive tissue changes and scarring in several other CNS pathologies, spinal cord injury represents a particularly striking example where wound repair is inefficient and injury-induced pathological changes are insurmountable.

There are several likely contributing factors to these regional and injury-specific differences in CNS scarring. Cell types involved in scarring in different CNS regions are phenotypically different^19^. There are also differences in the levels of neuroinflammation and astrocyte activation after brain and spinal cord trauma, with increased expression of inflammatory cytokines and damage-exacerbating leukocytes^20–22^ and more abundant and widespread astrocytosis in spinal cord injuries compared to brain injuries^14,20^. There are also differences in ECM composition and distribution between brain and spinal cord pathologies^23–25^. In this Review we specifically focus on scarring following spinal cord injury, and discuss the dynamic cellular and extracellular interactions that culminate in a hostile scar environment with limited capacity for repair.

### Defining the spinal injury scar

**Fig. 1:**
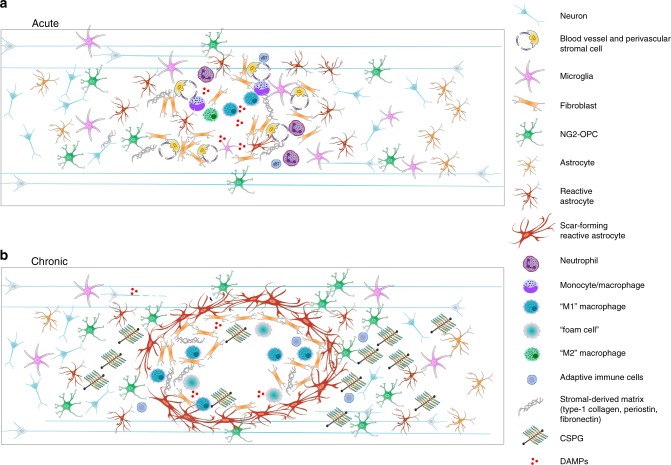
Cellular and extracellular composition of the spinal injury scar. Traumatic spinal cord injury triggers a complex cascade of events that culminate in the spinal injury scar which consists of multiple cell types as well as extracellular and non-neural components. **a** In the acute post-injury phase (0–72 h), cell death and damage lead to release of a number of cellular and blood-derived DAMPs (damage associated molecular patterns). These are powerful activating and inflammatory stimuli for stromal cells, astrocytes, NG2 + OPCs and microglia. Fibroblast-like cells proliferate from perivascular origin. Activated cells increase deposition of extracellular matrix molecules such as chondroitin sulfate proteoglycans (CSPGs) and stromal-derived matrix. Circulating immune-responders (neutrophils, monocytes) are recruited, their relative expression of cytokines, chemokines and matrix metalloproteinases becomes shaped by the early injury environment, and a mixed immune cell phenotype (M1, pro-inflammatory; M2, pro-resolving) is initially adopted. This becomes increasingly proinflammatory. **b** In the chronic spinal injury scar, monocyte-derived macrophages/microglia adopt a predominantly M1 phenotype. Rather than entering a phase of resolution, responding innate immune cells present DAMPs to circulating adaptive immune cells and pathology spreads. Reactive astrocytes hypertrophy, upregulate expression of intermediate-filament associated proteins and secrete matrix CSPGs. Fibroblast-like cells contribute to fibrotic tissue remodelling and deposition of stromal-derived matrix. Innate immune cells become unable to process cellular and matrix debris effectively and become synonymous with lipid-rich foam cells. Scar-forming reactive astrocytes organise into a barrier-like structure which separates spared tissue from a central region of inflammation and fibrosis where wound-healing fails to undergo resolution. In most mammalian species a chronic cystic cavity develops. Wallerian degeneration of injured axonal projections contributes to continued extracellular deposition of axonal and myelin debris, which is ineffectively processed by immune cells, and along with CSPGs, acts to inhibit neuronal regeneration and neuroplasticity long-term

The scar that forms after a spinal cord injury is generally considered to have two distinct components: the lesion core, which is primarily composed of stromal-derived fibroblasts and inflammatory immune cells, and the lesion border, or penumbra, which surrounds the core and is primarily composed of hypertrophic astrocytes^26–28^. The term glial scar has historically been used to describe the astrocyte border component of the scar^29–31^, although some investigators use the term more widely to reflect the entire lesion including both glial and non-neural components^32^. Other distinctions have been made between the glial scar and the fibrotic scar^27,33^, and specific components of the lesion (the core, the astrocyte border, and surrounding tissue) have recently been referred to as lesion-related tissue compartments^31^. While these distinctions are valid, and scar components undoubtedly become spatially compartmentalized in a chronic scar, these components are nonetheless interlinked, and evidence suggests that there may be temporal dependence and bi-directional cross talk between them. Furthermore, the scar should not be considered as an isolated component of spinal injury pathology since it is shaped by processes of inflammation and tissue and matrix remodelling. Here we use the term spinal injury scar, which encompasses both cellular and extracellular components across the lesion core, lesion border and surrounding penumbra (Fig. 1). Below, we discuss the events after the initial spinal cord trauma that contribute to spinal injury scar formation and maintenance.

### Acute signalling events post-injury

**Fig. 2:**
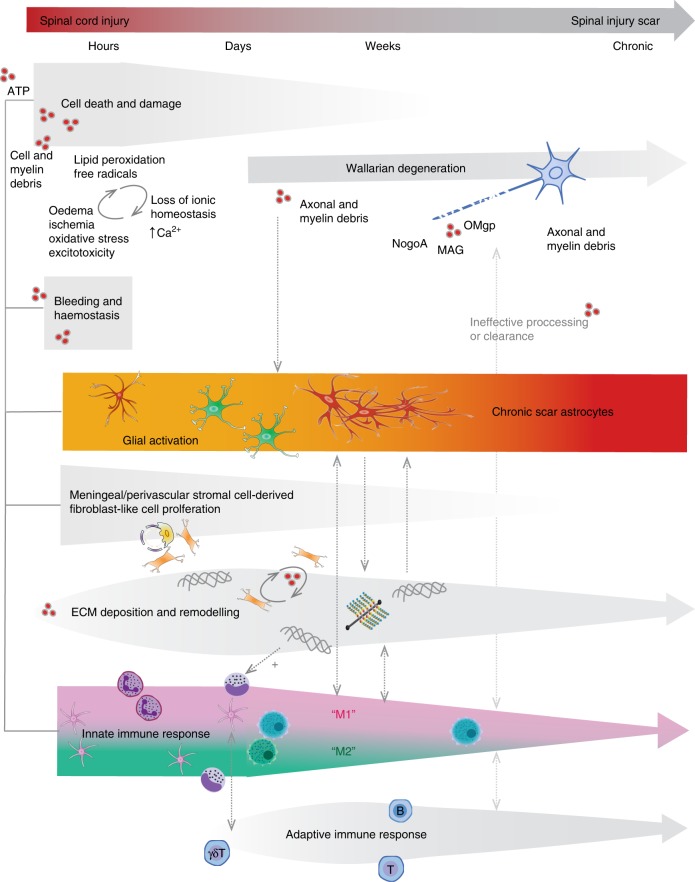
From injury to scar: time course of progressive scar pathology showing interlinked relationships between different components of the spinal injury scar. Following traumatic spinal cord injury, acute cell death and damage triggers release of cell-derived and blood-derived DAMPs, ATP release, dysregulated ionic homeostasis oxidative stress and excitotoxicity, which represent potent stimuli for triggering glial cell activation, stromal cell proliferation, deposition of extracellular matrix (ECM), and recruitment of circulating innate immune cells. Within a few days following injury, monocyte-derived macrophage/microglia adopt a predominantly M1 phenotype which do not favour resolution and tissue remodelling becomes fibrotic. Proinflammatory innate responders also present DAMP-derived antigens (such as MBP) to T and B-cells. B cells, in turn, may present antigens to T-cells, triggering their expansion. During this time, reactive astrocytes proliferate, hypertrophy and overlap in order to isolate this zone of non-resolving pathology from spared tissue. They also secrete matrix CSPGs, which are known to downregulate neuronal plasticity. Wallerian degeneration of degenerating axonal tracts contributes to continued deposition of axonal and myelin debris, which is ineffectively processed by immune cells and leads to the deposition of myelin-associated molecules (MAG, Nogo, OMgp) which are known inhibitors of neuronal regrowth. Ongoing Wallerian degeneration at later post-injury stages further triggers gliosis and neuroinflammation. Dashed grey arrows show cross talk between different components of the spinal injury scar, which is usually bidirectional. For example, CSPGs released by reactive astrocytes are thought to activate receptors on macrophages/microglia to induce a proinflammatory phenotype and in turn increasing inflammation induces further astrocytic reactivity and CSPG deposition. Fibroblast-like cells also synthesise type 1 collagen, implicated in the induction of astrogliosis and further deposition of matrix molecules. Cross talk between the innate and adaptive immune response also propagates inflammatory pathology and further influences glial activation and CSPG production. The dynamic interactions between inflammation, dramatic tissue and ECM remodelling and reactive cellular and extracellular changes drive the progressive, propagating pathology that culminates in the spinal injury scar

The majority of spinal cord injuries feature a contusive component, where compromised spinal canal shape or volume causes physical deformation of spinal cord tissue^2^ (rarer presentations include sharp penetrating trauma to the spinal cord, or purely ischaemic lesions following vascular compromise). Tissue deformation transmits shearing and compressive forces on axons and blood vessels and initiates a cascade of pathological processes. Below, we discuss these processes, focusing on changes occurring immediately after the initial spinal cord trauma up to one day post injury. These acute post-injury events signal the beginning of the injury cascade, which culminates in chronic pathology and scarring (summarized in Fig. 2).

Vascular trauma leads to haemorrhage, and accumulating blood sera increases tissue colloid osmotic pressure, causing local oedema and swelling^34^. This damage, along with vasospasm of spared vessels, leads to tissue ischaemia. ATP released from damaged or metabolically-compromised cells acts on purinergic receptors to induce microglial chemotaxis towards the injury zone to protect against spread of damage^35–38^. ATP gradients are also propagated via connexins^39^. Oligodendrocytes, oligodendrocyte precursor cells (OPCs), microglia and astrocytes express a heterogeneous mix of P2 receptor subtypes, and respond reactively to increasing levels of ATP following trauma^40,41^.

Subsequently, tissue reperfusion induces further oxidative stress, glutamate release and death of neighbouring neurons and glia via excitotoxicity^42^. ATP release, dramatic loss of cellular and extracellular ionic homeostasis and excessive calcium levels results in the activation of calpains, phospholipase A_2_ and lipoxogenase. This is followed by the generation of bioactive lipid mediators and free radicals^43^. Progressive oxidation of fatty acids in cell membranes and myelin (lipid peroxidation) occurs. Furthermore there is feed-forward propagation of the injury and cell reactivity, as bioactive mediators potentiate ATP-mediated calcium increases in glia^40^ and additional blood vessel endothelial cell damage results in an expanding zone of haemorrhagic necrosis^44^.

Concomitantly, haemostasis is the first stage of wound healing: endothelial cell trauma results in platelet adhesion and activation, the coagulation cascade, and thrombin-mediated conversion of fibrinogen to fibrin, to form a clot. The onset of hemostasis^45^ is associated with reactive changes in resident glia and represents a potent inflammatory stimulus. Platelets themselves are an abundant source of inflammatory peptides and protein mediators and release cytokines, chemokines and eicosanoids which readily communicate with resident spinal cord cells and non-resident leukocytes. These signals cause rapid neutrophil infiltration within an hour (peaking within 24 h), which secrete MMP9, a type IV collagenase which acts on basement membranes to further permeabilise the blood brain barrier^34^. Furthermore, cellular and extracellular factors (which are rapidly induced as a result of trauma, injury expansion and necrosis) constitute host-derived danger signals^46^. Damage associate molecular patterns (DAMPs) are sterile inflammatory stimuli (such as ATP, HMGB1, IL33) which activate prototypical pathogen recognition receptors (TLRs, NLRs, signalling via MAPK, NFΚB) to induce secretion of proinflammatory cytokines and chemokines from both neurons and glia, compounding reactive gliosis and acting to recruit circulating immune responders^47–50^.

### Non-resolving pathology

Non-resolving pathology results in incomplete tissue repair and formation of a scar. Each cell type that contributes to this pathology, and its respective phenotype, is inherently linked to the environment it finds itself in. This environment constitutes a milieu of other resident and non-resident cells, the signals they transmit and the biochemical and biophysical properties of the extracellular environment in which they reside. Below, we discuss cellular and extracellular changes occurring in the sub-acute period, from days to several weeks after the initial spinal cord trauma, and their contribution to the spinal injury scar. Cellular and extracellular components of the spinal injury scar in the acute and chronic phases of spinal cord injury are depicted in Fig. 1.

## Cellular components of the scar

### Astrocytes

Astrocytes become reactive following spinal cord injury. The degree of reactivity is influenced by a number of cell-surface receptors for DAMPS and proinflammatory cytokines and chemokines^51^, and ranges from temporary changes in gene expression and cell morphology to significant hypertrophy, spatial rearrangement and proliferation (collectively termed astrogliosis)^52,53^. Additionally, diverse astrocyte subsets and phenotypes may exist following spinal cord injury (Box 1). Astrocytes are also known to dynamically switch from reactive to quiescent when transposed from injured to naïve spinal cord^54^. Reactive astrocytes densely populate the borders of the injury epicentre, hypertrophy and strongly upregulate the expression of intermediate filament proteins such as GFAP, nestin and vimentin^53,55,56^. This corresponds with elongation and extension of overlapping processes (unlike parallel and radial processes found throughout normal CNS tissue architecture) and the organization of astrocytes into a barrier-like structure^57^.

This barrier is reinforced by proliferation and organization of a local astrocyte population at the injury border, thought to be mediated via STAT-3 dependent signalling^58,59^ and leucine zipper-bearing kinase (LZK, MAP3K13) expression^56^. Ependymal cell-derived astrocyte-like progeny contribute to this population of astrocytes in certain spinal injury models^60^, athough evidence suggests this contribution is minor following contusive injuries^61^. Tight linking of astrocytes at the injury borders is associated with reformation of the glia limitans and the containment of immune cells and fibroblast-like cells within the injury epicenter, via ephrin-mediated cellular adhesion^62,63^. Thus, a population of reactive astrocytes act to spatially isolate damage and fibrosis from spared tissue.

An overlapping population of astrocytes are further associated with the chronic maintenance of this structure. These are referred to as scar forming astrocytes^64^. Though astrocytes alone are not responsible for the formation of a scar, they are major cellular players activated and maintained during post-injury pathology, inflammation and tissue and matrix remodelling. Alongside other cells and extracellular factors, astrocytes shape the scar cellular and extracellular milieu sub-acutely and chronically.

### Fibroblast-like cells

Fibroblasts are ubiquitous in peripheral connective tissues and organs and are the principal generators of stroma, including the ECM. By contrast, under normal conditions within the CNS, fibroblast-like cells are mostly associated with the vasculature, and contribute only to the basal laminae. However, injury to the spinal cord induces a significant fibroblast response which produces matrix components. These matrix components may inhibit neural regeneration directly, and promote prolonged tissue remodelling via interaction with inflammatory cells (detailed below). These stromal elements become spatially compartmentalized by surrounding reactive astrocytes to form the fibrotic core of the spinal injury scar.

Fibroblasts proliferate from meningeal cells if the dura is compromised^27,33^ and can derive from perivascular cells in rats^65^ and mice^63^ following contusion injury. There is some evidence that fibroblast-like stromal cells derive from pericytes; the PDGFRβ^+^ Glast^+^ perivascular cell population, termed type A pericytes, proliferate in response to injury and contribute to fibrotic scarring^66^. Preventing Glast1+ cell proliferation leads to failure of wound sealing, exacerbated lesion volume and decreased matrix deposition^66^, whereas moderate reduction of pericyte-derived fibrosis was found to reduce scar pathology and confer functional recovery^67^. However, whether this is a truly separate population or whether it overlaps with known cell types (such as Glast+ astrocytes in the glia limitans) is unclear.

An increase in type-1 pericytes (distinct from those described above) has also been described, following a non-contusive dorsal funiculus lesion using a nestin-GFP/NG2-DsRed transgenic mouse line^68^. However, NG2 is also a marker of OPCs, Schwann cells and macrophages following spinal cord injury^69^. Thus, there is some ambiguity as to the response of pericytes following injury, although collectively there is evidence that fibroblast-like cells are derived from a perivascular PDGFRβ+ origin^63,66,70^.

### Oligodendrocyte precursor cells

NG2+ OPCs become reactive after spinal cord injury, have a significant proliferative capacity and are spatially intermingled with other reactive glia at the injury border^71,72^. Two main contributions of OPCs within the spinal injury scar environment have been described, with seemingly opposing roles. OPCs contribute to remyelination, either by oligodendrogenesis or through differentiation into remyelinating Schwann cells^73–75^, and also hypertrophy and increase expression of NG2, a proteoglycan thought to mediate entrapment of neurons^76^. However, the roles of OPCs in the spinal injury scar have been obscured experimentally by overlap of markers with other cells. Alongside NG2, PDGFRα, two ganglioside antigens, and a cyclic nucleotide phosphodiesterase (also thought to be expressed in microglia), OPCs also express the traditional astrocyte marker GFAP, and may differentiate into a de novo population of astrocytes in the scar^72,77^. Additionally, both PDGFRα and NG2 are also thought to be expressed by at least one type of perivascular/pericyte-type cell^71^. The use of fate mapping transgenic mouse lines, such as those expressing Cre recombinase under control of the PDGFRα promoter/enhancers^73–75,78^, alongside inclusion/exclusion of specific markers, will further define their role and contribution to scarring after spinal cord injury.

### Resident microglia and innate and adaptive immune cells

By 24 h after spinal cord injury, blood-derived monocytes are recruited into the lesion. Upon extravasation, DAMPs and the associated reactive and inflammatory environment shape their differentiation phenotype. Meanwhile, resident microglia retract cellular processes and become morphologically indistinguishable from infiltrated monocyte-derived macrophages. Until recently, microglia and macrophages were only distinguishable using relative gene expression of CD45 (macrophages being defined as CD11b^+^, CD45^high^ and microglia CD11b^+^, CD45^low^) or chimeric models. Specific, or enriched, markers for microglia have now been discovered, including transmembrane protein 119 (Tmem119)^79^, P2ry12 and Fc receptor-like S (FCRLS)^80^. Tracking resident microglia via a genetic strategy has shown that spared microglia proliferate and repopulate the lesion core alongside monocyte-derived macrophages^38^ and recent availability of Tmem119 reporter mice^81,82^ will further elucidate the role of microglia in spinal injury scarring.

A monocyte-derived or microglia-derived macrophage phenotypic spectrum exists, from pro-inflammatory (termed M1, secreting TNFα, IL-1β, IL-6, IL-12) to pro-repair (termed M2, secreting IL-10, IL13). Following spinal cord injury, there is initially a mixed M1/M2 response^83^. The release of pro-inflammatory cytokines at the injury site further mobilizes resident and blood-derived cells to phagocytose debris^84,85^ and affects the phenotype of other nearby resident cells.

The adaptive immune system also plays a role. After spinal cord injury, the recruitment of γδT cells, and production of proinflammatory IFNγ occurs within 24 h following injury^86^. Other adaptive immune system components are recruited by 7 days, and contribute to non-resolving trauma-induced autoimmunity^87^. Leukocytes present DAMP-derived antigens (such as MBP) to T and B-cells. B cells, in turn, may present antigens to T-cells, triggering their expansion. Furthermore, B-cells differentiate into plasma cells synthesizing auto-antibodies, further fuelling a feed-forward immune response^88^.

Unlike conditions in which successful wound healing occurs, there is no effective resolution to cellular recruitment and inflammation after spinal cord injury. Monocyte or microglia-derived macrophages remain in the injured spinal cord indefinitely^89^. Macrophages maintain 45% of peak activation months after injury^90^ and their phenotype does not undergo the switch from pro-inflammatory to pro-repair associated with the next phases of wound-healing in other organ tissues^91^. The early arginase1+ (M2-like) differentiating infiltrating population is not maintained^83^ and the spectrum of innate immune cell activation phenotype is predominately M1 polarized. Adaptive immunity is non-resolving, whereby lymphocytes remain indefinitely in the spinal injury scar.

### Interaction between cell types

The response of astrocytes, OPCs, microglia and infiltrating innate and adaptive immune cells is continually influenced by one another and the tissue environment (Fig. 2 depicts the time course of reactive resident and non-resident cell recruitment and activation, and their cross-linked interactions which lead to the chronic spinal injury scar). There are multiple direct and indirect cellular interactions mediated by cytokines and chemokines which underlie this (alongside interactions which occur via ECM components, discussed below). Perivascular astrocyte endfeet are an integral part of the endothelial blood spinal cord barrier, and thus astrocytes are in a position to regulate the magnitude of leucocyte recruitment. Astrocyte expression of Socs3^92^ or NFKB^93^ increases monocyte infiltration to the lesion epicentre. In addition, resident and infiltrated microglia/macrophages express a number of receptors for proinflammatory chemokines and cytokines released by reactive astrocytes (such as IL-6, IL1β, CCL2), contributing to a cell signalling environment which potentiates M1 polarization. In turn, astrocytes express receptors for a number of inflammatory mediators released by immune cells (including IFNγ, IL6, IL1β, TNFα), inducing extensive astrocyte reactivity and astrogliosis^53,94^. Thus, there is an intimate link between astrocytes and resident and infiltrating immune cells during formation of the spinal injury scar. Astrocyte-fibroblast interactions have been shown to spatially compartmentalize the fibrotic core^62^ and recent evidence suggests that microglia may provide an additional interface between these cells, which the authors term the microglial scar^38^.

In addition to direct cellular cross-talk, almost all parenchymal cells express receptors for a multitude of signalling molecules present in the external injury microenvironment and indirectly affect cell activation and phenotype of surrounding cells. Within this, there are canonical regulators. For example, abolishing Wnt signalling in OPCs has been shown to reduce monocyte accumulation and astrocyte hypertrophy^95^. Furthermore, neurons themselves are directly contacted by cells in a manner which inhibits reestablishment of neuronal connectivity. Proinflammatory ED1/CD68+ macrophages induce axonal dieback upon contact^96,97^, NG2+ OPCs mediate neuronal entrapment^76^, and perivascular Glast 1+ cells are also directly contacted by stalled axons^67^.

Importantly, the reactive cellular responses after spinal cord injury are not effectively resolved and many aspects are maintained chronically. Macrophages retain activity long-term with maintained M1 like characteristics. Glia continue to be reactive in regions of tissue spared by injury and in areas remote from the site of trauma^98^, partly in response to Wallerian Degeneration^99,100^. Proximal to the lesion, glia remain hypertrophic, forming a compacted astroglial scar border in which spared tissue is permanently isolated from a zone of unresolved pathology, fibrosis and tissue loss. This zone is not effectively repopulated by neurons or glia and, in most mammalian species, develops into a chronic cystic cavity (Fig. 1).

Box 1 Phenotypic diversity and plasticity of astrocytes: emerging evidence from brain and spinal cord injury There is increasing data available on the cellular profile and phenotypic diversity of astrocytes after injury. Phenotypic nomenclature comparable to that adopted in characterization of macrophages/microglia has been used for the transcriptional profiling of cultured astrocytes isolated from the CNS under different injury conditions. Ischemic injury in the brain (modelling stroke) leads to a trophic A2 polarization state. By contrast, activated microglia from models of neurodegenerative and neuroinflammatory diseases and traumatic optic nerve crush injury release factors such as IL1α, TNF and complement component subunit 1q (C1q) which induce a neurotoxic state in A1 astrocytes^55,94^. In the optic nerve, there is evidence that axotomy-induced A1 astrocytes, in turn, kill axotomized neurons. In these studies, microglial-derived mediators were necessary and sufficient to induce an A1 phenotype. Whether this occurs following spinal cord injury is, as yet, unreported.

Genetic profiling of reactive astrocytes and scar-forming astrocytes following spinal cord injury (isolated by laser-capture microdissection at 7 and 14 days post injury, respectively) has recently been reported. Reactive astrocytes were associated with selective upregulation of *Nes, Ctnnb1, Axin2, Plaur, Mmp2*, and *Mmp13* whereas scar-forming astrocytes selectively upregulated *Cdh2, Sox9, Xylt1, Chst11, Csgalnact1, Acan, Pcan* and *Slit2*^54^. Furthermore, using these genes as population markers, FACs isolated nestin-GFP+ reactive astrocytes were found to convert to a naïve phenotype following transplantation into uninjured tissue but become scar-forming when transplanted into injured tissue, an effect thought to be mediated by a N-cadherin-dependent interaction with type 1 collagen^54^. Thus, astrocytes are able to display phenotypic plasticity, and tissue environment is a crucial influence over cellular behaviour.

The GFAP-RiboTag mouse can be used to perform high-throughput RNA sequencing on astrocytes following injury and probe the effect of particular genes in the astrocyte response^108^. Two weeks after a spinal crush injury RNA-seq revealed differential expression of over 6000 genes in astrocytes, changes described as congruent with prior transcriptomic analysis following ischemic stroke lesion^55,108^. However, whether the astrocytes sampled here represent A2-like trophic astrocytes has not been ascertained.

There are some discrepancies between gene expression findings using these different methodologies. For example, of the eight genes associated with scar-forming astrocytes isolated using laser capture microdissection^54^, the RNA-seq dataset only supports increased expression in one of these (*Xylt1*)^108^, whereas four of the six genes ascribed to reactive astrocytes^54^, are increased (*Nes, Axin2, Plaur, Mmp2*)^108^. Spatial differences in sample selection may contribute to these disparities. Techniques such as 3D intact-tissue RNA sequencing^182^ may overcome this problem in the future. Indeed, further characterization of spatio-temporal phenotypic diversity and plasticity would aid research into how astrocyte phenotype and scar progression may be modified by changes to matrix components or perturbation of the immune response. Such methods, alongside new purification techniques for in vitro analysis, will likely provide increasingly nuanced understanding of the relationship between astrocytes, microglia, non-resident immune cells and the tissue environment^79,185^.

## Extracellular components of the scar

The CNS ECM is rich in glycoproteins and proteoglycans. Hyaluronan forms a backbone for the attachment of tenascins and sulphated proteoglycans, stabilised by link proteins. This is arranged either diffusely in the interstitial space or more densely assembled around the cell soma of particular neuronal subtypes (as perineuronal nets), or around axonal nodes of ranvier or synaptic boutons. These structures confer neural stability, localizing molecules such as CSPGs, which effectively restrict large-scale plasticity following a critical period in development^101^. Following injury, resident glia and stromal cells, which do not normally contribute parenchymal matrix, begin to contribute matrix components, and extracellular DAMPs are present in both sub-acute and chronic phases.

A vast number of ECM molecules undergo differential regulation following spinal cord injury (for a large scale validation see ref. ^50^) and many of these play a role in neuroprotection or spontaneous repair and are not refractory to recovery. Fibrous matrix forms a seal or tissue bridge between retracting lesioned parenchyma. This is particularly apparent in injuries where spinal tissue is rendered non-continuous, for example, following hemisection or transection. Fibroblast-derived collagenous matrix is also a major component of the ECM^33^ (and in vivo ablation of fibroblasts compromises tissue integrity following injury)^66^. Basal laminae is restored via matrix deposition of collagen VI, nidogen, fibronectin and laminin, which are traditionally neuronal-growth permissive molecules. However, in the context of spinal cord injury, such ECM molecules are also implicated in pathological tissue remodelling or inflammation. For example, fibronectin, matrix glycoprotein tenascin C and hyaluronan fragments also act as endogenous TLR ligands and represent DAMPs^48^.

### Extracellular components fuel fibrosis and scarring

Initial and expanding secondary pathology generates a large amount of cellular and myelin debris, sustained by longer-term Wallerian degeneration, oligodendrocyte apoptosis and demyelination. The presence of debris, and its breakdown products, supports an ongoing foam-cell-like macrophage phenotype^102^ where ineffective phagocytosis and lipid processing means extracellular stimuli are maintained^85^ and are presented to adaptive immune cells, which contributes to a non-resolving auto-immune response to injury^87^. Thus, the extracellular environment is undergoing both chronic inflammation and glial reactivity, associated with aberrant tissue remodelling and matrix deposition. There is increasing understanding as to how these processes are intertwined.

A recent study demonstrated that perivascular PDGFRβ+ cells (described as pericytes) upregulate expression of the ECM molecule periostin, which in turn upregulates TNFα expression from infiltrating monocyte-macrophages and leads to proliferation of PDGFRβ+ cells, type-I collagen deposition and fibrosis^70^. Perivascular-derived type-1 collagen has also recently been implicated in linking fibrosis and astrogliosis, where an N-cadherin dependent interaction between extracellular type-1 collagen and astrocytes was found to induce scar-forming astrogliosis in mice^54^. Thus, there is expanding evidence supporting a role for perivascular Col1α1-cell derived fibrotic matrix following contusion injury in mice^63^. PDGFRβ+, fibronectin-rich fibrotic matrix deposition is also observed in rats in the peripheral rim of the cavity and outlining blood vessels^103^, suggesting a somewhat conserved contribution despite differences in cavity formation between mice and rats. Matrix deposition of type-1 collagen has also been described as a perivascular-fibroblast-derived scaffold for neoangiogenesis^65^. Defining the role of collagen in fibrosis and angiogenesis in spinal cord injury requires further study.

### Extracellular components are inhibitory to neural regeneration and plasticity

In addition to an ongoing DAMP role for myelin debris, myelin-associated molecules confer extrinsic inhibition to neurons. These include Nogo A, myelin-associated glycoprotein (MAG) and oligodendrocyte myelin glycoprotein (OMgp). Nogo-A is a potent inhibitor to neural plasticity and regeneration following spinal cord injury^104^, preventing axons from overcoming the spinal injury scar environment. Transmembrane receptor complexes are identified, converging on the canonical RhoA/ROCK signalling pathway, resulting in destabilization of the actin cytoskeleton and local arrest and collapse of growth cones^105^. Furthermore, CSPGs (Box 2) are upregulated by reactive glia following spinal cord injury both perilesionally and at distal spinal segments^98,106^ and are associated with decreased plasticity and abortive regeneration^107^. Recent evidence suggests that scar-forming astrocytes express brevican, and NG2, though not aggrecan^108^. There is some evidence that core CSPG proteins are inhibitory to neuronal growth^109^ but CS-GAG chains are known to confer significant inhibition following injury as their removal promotes anatomical and functional recovery following spinal cord injury^110^. Membrane-bound receptors to CS-GAGs, reported to mediate inhibition, include RPTPσ^111,112^, leukocyte common antigen-related phosphatase (LAR)^113^, NgR1 and NgR3^114^. Signalling pathways implicated have convergence with those of Nogo and other myelin inhibitors and include the Rho/ROCK pathway, activation of which is partly via PKC^115^ and EGFR^116^ and coupled to Akt/GSK-3 activation^117^. CSPGs are also thought to inactivate neural intergrins^118^ and localise upregulated inhibitory guidance molecules such as semaphorin 3A^119^. Thus, the injured spinal cord extracellular environment contains molecules which restrict neurite outgrowth and plasticity, and these are further upregulated and concentrated in the spinal injury scar and represent therapeutic targets^120^ (discussed below).

Box 2 Structure of chondroitin sulfate proteoglycans (CSPGs) CSPGs are proteoglycans (PGs) consisting of a core protein with at least one covalently attached chondroitin sulfate glycosaminoglycan (CS-GAG) side chain (see figure). CSPG subtypes most commonly studied with respect to the inhibitory CNS environment include lecticans (aggrecan, versican, neurocan and brevican), the transmembrane protein NG2, phosphacan (transmembrane or soluble) and the small leucine-rich proteoglycans decorin and biglycan. There are also multiple less-well studied CSPGs revealed by proteomics analysis of scar matrix^50^. Lecticans are the most abundant CSPGs in the spinal injury scar and also feature globular domains: the G1 N-terminal domain and G3 C-terminal domains are important in their interaction via link-protein with hyaluronan (the backbone glycoprotein of the CNS matrix) and also tenascin, thus they are involved in matrix crosslinking. Core PGs undergo post-translational modification in the endoplasmic reticulum and golgi, catalysed by a number of enzymes. At particular serine residues a tetrasaccharide linking region is formed by sequential addition of xylose by xylosyl transferase, two galactose molecules by β1,4-Galactosyltransferase-I then β1,3-Galactosyltransferase-II and a one GlcA residue via β1,3-glucuronyltransferase I to form the linker GlcAβ1–3 Galβ1–3 Galβ1–4 Xylβ1–O-Ser. The following addition of GalNac by a GalNac transferase I is crucial to initiate synthesis of the chondroitin sulfate backbone. If, at this point, N-acetylglucosamine (GlcNac) is added rather than GalNac, synthesis of the heparan sulfate backbone is initiated^186^. CS-GAG chain polymerisation is the process by which alternating residues of GalNac and GlcA are then added to the proteoglycan linker region by the alternating activity of a GlcA transferase II and a GalNac transferase II. There are six actual enzymes identified which confer this glycosyltransferase activity. Alongside CS-GalNac transferase I and II, combinations of enzyme complexes of chondroitin synthase 1, 2 and 3 and chondroitin polymerising factor (ChPF) mediate GAG polymerization.

## Biomechanical properties of the scar

Relatively little attention has been given to how the biomechanical environment of the injured spinal cord affects repair^121^. Cells are highly mechanosensitive and changes in the elastic properties of the environment alone can induce differentiation and migration^122^, and during development mechanical gradients guide axon pathfinding^123^. Astrocytes that are cultured on less compliant, stiff substrates anatomically resemble reactive astrocytes, displaying hypertrophy and elongation, with stiffness of CNS implants correlating with induction of reactive astrocytosis^124^. Integrin-mediated links between fibrillar type 1 collagen (known to be stiffer in other tissues) and astrocyte reactivity are emerging^54^, which may be influenced by mechanotransduction. Atomic force microscopy has been used to characterize the spatiotemporal elastic stiffness properties of spinal cord tissue over 1 to 3 weeks following dorsal column crush lesion^125^. At these early post-injury time points, tissue softened in areas corresponding to scarring and ECM deposition. This was somewhat surprising because scar tissue outside the CNS is typically stiffer than surrounding healthy tissue^126^, and was attributed to a lack of collagen-1 and loss of CNS myelin in these types of injuries (both of which scale with tissue stiffness)^127,128^, as well as the cellular composition of the scar (glial cells are softer than peripheral scar myofibroblasts). It will be important to further characterize these properties in more clinically relevant contusion-type injuries and in chronic injuries with established scar tissue, particularly given the increasing evidence of a role for collagen-1 in chronic contusive injuries^54,63,129^.

## The semantics of defining the scar as good or bad

There has been some recent debate in the field on whether the scar is good or bad in terms of recovery from injury^108,130–132^. We propose that these two different viewpoints reflect different interpretations of data which is, in fact, largely in agreement. Both historical and newer findings support a long-established principle, that the spinal injury scar performs dual, and seemingly opposing, roles; to protect tissue, and to inhibit repair.

As previously introduced, the classical description of the spinal injury scar is one which considered the astrocyte-rich injury border alone, termed the glial scar. Early observations of dense glial reactivity at the site of CNS lesions led to the hypothesis that the astrocytic scar inhibits axon regeneration, perhaps by forming an impenetrable barrier to axonal extension^30^. An inhibitory role for the scar and scar-associated molecules has been well documented ever since (as discussed above and^26,27,106,107,110,111,116,133,134^). However, it has also long been acknowledged that the astrocytic scar has an important protective role in enabling the separation of healthy tissue from pathology following injury^135–137^. Thus, for decades the dual notions that the scar is associated with failed axonal re-connectivity (inhibitory) and also involved in a wound-healing response (protective), have existed.

In addition, as discussed above, there is now increased appreciation of the multiple cell types, beyond astrocytes, which contribute to spinal injury scarring, together with extracellular and non-neural components. This renders the term glial scar an insufficient descriptor. The multifaceted nature of the scar should be considered when interpreting experimental approaches which prevent scar formation. For example, a number of transgenic loss of function experiments have been performed to specifically investigate the role of astrocytes following spinal cord injury, including formation of the glia limitans and continued presence in the chronic scar. Early evidence suggested that double knockout mice for the intermediate filament proteins GFAP and vimentin (but not either protein alone) develop a less dense glial scar, with greater haemorrhaging, fibrosis and presence of debris following lesion to the dorsal funiculus^138^. Similarly, conditional ablation of proliferating (scar-forming) reactive astrocytes following injury increases edema, inflammation, oligodendrocyte death, tissue loss, demyelination and functional deficits^139,140^. Furthermore, deletion of SOCS3 or STAT3 in astrocytes results in lesion expansion, cell death and exacerbated functional outcome^58,92^. Thus, a number of studies have provided evidence for the importance of reactive astrocytes in preventing expansion of pathology into spared peri-lesional regions of the spinal cord. A recent extension of these studies utilized these deletion strategies to specifically assess the effects of reactive astrocytes on axonal regeneration^108^. The study revealed that spontaneous regrowth of damaged axons does not occur across a spinal crush injury following attenuation or ablation of scar forming astrocytes, despite boosting the regenerative state of these axons with a conditioning lesion and neurotrophin delivery^108^. This study claimed to reveal a new (and controversial) role of the astrocytic scar as being pro-regenerative. Our interpretation of these findings, however, is that they are largely consistent with previous studies^58,92,140^. A lack of axon regeneration after ablating reactive scar astrocytes does not necessarily mean that the glial scar aids axon regeneration. An alternative interpretation is that the regenerative boost afforded by conditioning lesions and neurotrophin delivery is not sufficient to overcome the prior-established lesion-exacerbating effects of preventing astrocytic scar formation. Instead, injured axons are presented with neural and non-neural components of the spinal injury scar, including NG2+ OPCs, inflammatory cells and CSPGs, all of which are known blockers of regeneration^110,111,116,133,141^. Indeed, greater dieback of axons from the injury site was observed in this study^108^, in line with an advancing wall of inhibitory factors no longer contained in the fibrotic lesion core. Thus, rather than overturning an old dogma, this study used elegant genetic tools to demonstrate an important role for scar-forming astrocytes in tissue protection following traumatic spinal cord injury, supporting previous observations^58,92,139,140^ and confirming the early hypothesis postulated by Gopal D. Das: “If, by some means, glial scar formation could be completely eliminated, most of the atrophying axons still might not show regeneration, and the spinal cord would be continuously invaded by loose connective tissue and other foreign materials and organisms while undergoing a protracted degeneration”^135^.

Although astrocyte components of the spinal injury scar are well evidenced to be beneficial initially, they (and other scar elements) have been suggested to be detrimental at chronic post-injury stages^32,142,143^. However, astrocyte ablation at chronic timepoints indicates that astrocytes themselves are necessary for maintaining tissue integrity in chronic injuries, as functional outcome is negatively affected by their removal 5 weeks post injury^108^. Though their contribution to extracellular signalling may have both beneficial and detrimental elements.Thus, it will be important to further characterise the molecular profile of the spinal injury scar at precise time points after injury, both for the cell types involved in scarring (e.g. Box 1) as well as the extracellular components. For example, recent proteomics studies of subacute (1-2 week) and chronic (8 week) spinal injury tissues have revealed differential expression of multiple growth factors and inhibitory and permissive ECM molecules at different post-injury stages^144,50^, requiring further study as to their specific roles in shaping the response to injury.

Thus, the dual nature of the spinal injury scar has long been known, yet continues to be reviewed, revisited and reinterpreted^19,64,92,108,132,142,143,145^. Rather than focus on good versus bad, perhaps efforts would be best directed at understanding and targeting specific aspects of the scar to aid recovery. For example, it may be beneficial to target components of the scar which are non-permissive to regeneration or plasticity rather than removal of the astrocytes themselves, even at chronic time-points. Targeting diverse cell types and phenotypes, as well as extracellular and non-neural components should also be considered, as well as the timings of such interventions. These approaches will be discussed in the following section.

## Therapeutic strategies

Current experimental approaches for targeting the spinal injury scar attempt to either reduce scar formation, or to block inhibitory molecules associated with the scar, using a variety of surgical, pharmacological and genetic approaches. Some of these show promise for application in the clinic.

### Attenuating scar formation

Although preventing formation of the astrocytic component of the spinal injury scar impacts negatively on wound-healing (discussed above), as the mechanistic understanding of spinal injury scar pathology increases, a number of studies in preclinical rodent models have targeted mesenchymal or fibrotic-derived components in a bid to limit amplification of tissue damage. On a gross tissue scale, if the dura is breached, dural apposition and/or patching with another soft tissue material (duraplasty) is suggested to limit fibrotic and connective tissue deposition from meningeal-derived fibroblasts. Decompressive durotomy followed by dural allograft has been shown in rodent models to reduce scar formation and lesion volume, but if the dura is not replaced, lesion volume increases dramatically^146^ and indeed expansion duroplasty is performed alongside decompressive durototomy in clinical evaulations^147^.

The fibrotic components of the scar can also be targeted pharmacologically. Systemic administration of the microtubule stabilizing antimitotic agents taxol or Epothilone B leads to reduced migration of scar-forming fibroblasts and suppression of extensive scar formation, enabling axon regeneration and functional recovery^11,148^. This highlights the potential for repurposing of epothilones and taxanes that are already used in cancer treatment^149^. Inhibiting collagen synthesis using the iron chelators BPY-DCA (which inhibits prolyl 4-hydroxylase, a key enzyme of collagen IV synthesis) and cyclic adenosine monophosphate (cAMP, which inhibits meningeal fibroblast proliferation)^150,151^ reduces fibrotic scarring and promotes neuroprotection and long-distance axon regeneration^150^. Treatment with clinically approved ion chelator deferoxamine and inhibition of lysl oxidase, another key collagen biosynthetic enzyme, also improves outcome after partial spinal transection injuries in rodents^152,153^.

The gene expression profile or phenotype of astrocytes may also represent potential therapeutic targets with modulating effects on scar formation. For example, selective inhibition of NFΚB signalling in astrocytes reduces inflammation and is pro-reparatory in mice expressing a dominant negative ΚBα under the GFAP promoter^93^. However, this is difficult to target therapeutically. If current studies of astrocyte phenotype in the mouse brain (Box 1) translate experimentally to the injured spinal cord, newly identified gene/signalling targets and matrix targets (below) could be manipulated to depress propagating scar pathology. In vitro, application of human recombinant TGFβ3 (but not the removal of mediators IL1α, TNF and C1q) was able to rapidly reverse transformation from an A1 phenotype to an unreactive status^94^. Following optic nerve crush, this phenotypic conversion was demonstrated in vivo by delivery of antibodies to IL1α, TNF and C1q^94^. Manipulation of TGFβ3 was not reported in vivo, however multiple studies have targeted TGFβ1&2 following spinal cord injury^154^ to reduce spinal injury scar formation, so it would be interesting to know whether this is a TGFβ3-specific effect. Indeed TGFβ 1 and 2 are known to exert opposing effects to TGFβ3 on wound healing outside of the CNS, and human recombinant TGFβ3 has been utilized in clinical trials to promote dermal wound healing and scar reduction^155^.

### Targeting the extracellular matrix

Following spinal cord injury, the extracellular environment contains molecules which interact directly with neurons and other cell types (discussed above). Some of these are thought to augment pathology and extent of spinal injury scarring, while some directly inhibit the ability of neurons to overcome a scar environment and generate novel connectivity. Both are potential therapeutic targets.

Periostin is a secreted ECM protein which has recently been implicated in contributing to scar formation, via propagating fibrosis and inflammatory signalling^50,70^. Daily intraperitoneal injections with a mouse monoclonal antibody against periostin from 4 days to 2 weeks after injury was shown to reduce the extent of tissue pathology and scarring, which led to functional improvements in sensorimotor tasks following contusion injury in mice^70^. Generation of recombinant anti-periostin will allow this promising strategy to be further tested. Similarly, a recent study found that pathology could be attenuated within the first 2 weeks following spinal cord injury in mice treated with an N-cadherin neutralizing antibody, which blocked an integrin and N-cadherin dependent interaction between extracellular type-1 collagen and astrocytes and significantly attenuated astrocytic scar formation^54^. In this study the rapid behavioural recovery observed supports a neuroprotective role for neutralising n-cadherin, though this was not assessed directly. As above, these studies suggest that early fibrosis is an important therapeutic target for improving outcome after spinal cord injury. Furthermore, if A1/A2 polarisation factors^94^ are conserved following spinal cord injury, a known inhibitor of C1q is chondroitin sulfate A^156^, the mono CS-4 sulfated GAG, which raises interesting questions regarding additional roles of ECM proteoglycans and their sulfation epitopes following injury. Thus, matrix properties are a valuable means to tap into the plasticity of cell responses.

CSPGs (see Box 2) are known inhibitors of neuronal plasticity, present throughout the CNS ECM and highly concentrated in the spinal injury scar (discussed above). A number of experimental studies have reported functional improvement following spinal cord injury by genetic removal of enzymes critical for CS-GAG biosynthesis. This includes deoxyribozyme-mediated knockdown of xylosyltransferase-1 mRNA, the enzyme which catalyses GAG addition to the CSPG core protein^157^, conditional sox9 ablation^158^ and knockout of N-acetylegalactosaminyltransferase-1, the enzyme which catalyses the addition of the first GalNAc residue onto the tetrasaccharide link between the core PG and GAG^159^. Reports of therapeutically-applicable pharmacological approaches which recapitulate these effects are currently lacking.

Enzymatic strategies targeting CSPGs are a promising approach for spinal cord repair, due to their ability to render the ECM more permissive to neuronal plasticity and connectivity. Removal of CS-GAGs by the chondroitinase ABC (ChABC) enzyme has been widely demonstrated to have beneficial effects in enhancing axonal regeneration and neuroplasticity and promoting functional recovery following experimental spinal cord injury^110,160–163^. This effect has been replicated across multiple laboratories and in different species^164^, including mouse, rat, cat, and recently in primates^165^ and in a canine clinical model^166^. Furthermore, its use as an adjunct therapy can augment the benefits of other experimental therapeutics^167–169^. A gene therapy method of enzyme delivery, where host cells are themselves transduced to express the ChABC gene leads to extensive CS-GAG digestion, which results in reduced pathology and improved functional recovery following contusion injury to the thoracic^170^ and cervical^171,172^ spinal cord. Furthermore, widespread CSPG modulation achieved by viral delivery of ChABC promotes conversion of macrophages towards a pro-resolving M2 polarization state^170^ and drives an anti-inflammatory IL-10-mediated response^173^, which is likely to underlie reduced pathology. Thus, ChABC is a promising means to promote resolution of pathology as well as overcoming the inhibitory environment of the spinal injury scar. A recent study utilised a novel gene switch to enable controlled delivery of the ChABC gene and revealed that long term ChABC gene expression was required to elicit recovery of skilled reach and grasp functions, with recovery attributed to plasticity of descending systems^172^. Whether this viral ChABC approach will also have benefit when applied chronically is not yet established. However, recent work has demonstrated that a single injection of ChABC enzyme in the phrenic motor pool 1.5 years after unilateral cervical spinal cord injury was able to elicit rapid and robust recovery of respiratory function, restoring the ventilatory response to the paralysed hemidiaphragm^174^. Furthermore, chronic application of ChABC prior to transplantation of induced pluripotent stem cell-derived neural stem cells 7 weeks after a spinal compression injury led to reduced chronic-injury scarring, increased graft survival and improved limb function^175^. These studies highlight the potential for ChABC to unmask latent neuroplasticity and produce a microenvironment conducive to repair even within the chronic spinal injury scar.

Another enzymatic strategy that has recently been exploited for reducing CSPG inhibition is the mammalian enzyme Arylsulfatase B (ARSB, N-acetylgalatosamine-4-sulfatase), which removes C4S moieties specifically from CS-GAGs. In addition to being utilized in enzyme-replacement therapy for human mucopolysaccharidosis VI, ARSB administration has now been shown in one study to promote increased axonal sprouting and functional locomotor recovery following compression spinal cord injury in the mouse^176^. ARSB perhaps represents a more attractive, and more readily translatable, therapeutic prospect than a bacterial enzyme such as ChABC and certainly warrants further investigation, particularly given recent findings of eliciting enhanced axon growth in the injured optic nerve^177^. However, whether ARSB could elicit robust modulatory effects within the spinal injury scar microenvironment, comparable to ChABC, remains to be determined. Given the specificity of ARSB for 4S motifs, it may not be capable of the multi-modulatory effects that have been demonstrated for ChABC which include immune modulation, neuroprotection and neuroplasticity^164,178,179^.

Targeted modulation of CSPG receptor signalling via manipulation of the receptor PTPσ, has also proved to be a promising therapeutic prospect. The activity of the intracellular phosphatase domains of PTPσ are regulated via a conserved “wedge” structure which can occlude the catalytic domain, thus reducing phosphorylation activity and ability to signal downstream. Use of a membrane-permeable peptide mimetic of this wedge reduces PTPσ signalling following activation by ligands such as CSPGs. Systemic delivery of this peptide has been shown to enable recovery of locomotor and bladder function in rats following spinal contusion injury^180^ and the non-invasive nature of this approach means it is a potential candidate for rapid translation. Thus, approaches to overcome the inhibitory actions of CSPGs show collective promise in enabling beneficial alterations to the ECM associated with the spinal injury scar with positive effects in eliciting some functional repair.

### Future directions for therapy

The majority of approaches aimed at manipulating the spinal injury scar for therapeutic benefit have focused on modifying scar-associated ECM and targeting the synthesis, production and signalling of CSPGs. With the identification of cell subtypes that have opposing actions on tissue pathology, such as A1 neurotoxic vs A2 reparatory astrocytes^94^, there may be further opportunity to modulate astrocyte function or phenotype following spinal cord injury. These approaches will likely evolve as new markers are identified for delineating reactive astrocytes and microglia in different phenotypic states^181^, with increasing availability of astrocyte and microglia cell-specific sequencing data^54,79,108^ and with powerful emerging tissue sequencing technology^182^. Alternative approaches which may indirectly modulate astrocyte phenotype are also emerging, such as grafting specific stem cell populations which can influence host tissue cellular responses and drive astrocyte transformation to a permissive phenotype^132^. Additionally, with increased appreciation for the role of ECM molecules in affecting pathology and plasticity of cellular responses, novel targets may be identified with new matrix biology technology^183^. Conversely, the study of scarring mechanisms and ECM components in organisms that are capable of CNS regeneration may lead to the identification of pro-repair targets. For example, differential regulation of collagen XII within the scar matrix is one factor contributing to the pro-regenerative phenotype in zebrafish, controlled by Wnt/B catenin signalling^184^. Whether Wnt/B-catenin-mediated collagen XII production can be harnessed to render the mammalian spinal injury scar more permissive is not yet known. Finally, consideration should be given to scar biomechanics^121,125^ when designing therapies. It may be important to understand how pharmacological manipulations affect mechanobiology and further provide appropriate mechanical signals to optimize repair.

## Conclusion

The spinal injury scar is multifaceted. It contains more than just a reactive glial component and should be considered as a whole, since there is a complex interplay between multiple different cell types (glial cells, mesenchymal-derived cells, immune cells), their intracellular and signalling changes, and the extracellular environment. These processes modulate and feedback on each other. Altering the environment, for example using CSPG modulation with chondroitinase, can increase neuronal regeneration-associated gene expression and transcriptional changes^110,173^. Conversely, altering intracellular mechanisms can alter the inhibitory environment (for example microtubule stabilization with taxol or epothilone B leads to reduced fibrotic scarring^148^). Similarly, astrocyte-immune cell interactions are bidirectional, where an increasingly proinflammatory environment induces extensive astrogliosis^53^ and in turn, activated astrocytes release pro-inflammatory cytokines, chemokines and CSPGs, which can influence the magnitude of the inflammatory response^48^. The spinal injury scar has both beneficial properties (being essential for preventing spread of cellular damage) and detrimental properties (limiting new growth and tissue repair). This may be attributable to opposing phenotypes of reactive glial cells that form the scar border, given recent evidence in other CNS pathologies^94^. However, also important to note are the opposing roles of the scar matrix which contains beneficial molecules, required for formation of the glia limitans (which if not formed properly, can increase damage and worsen outcome) as well as molecules that are potent inhibitors of growth and neuroplasticity, such as CSPGs. Therapeutic strategies need to target detrimental aspects while preserving the beneficial properties of the spinal injury scar. Increased mechanistic understanding of the biological processes that propagate the non-resolving scar pathology is providing new therapeutic targets which may bring us closer to improving functional outcome following traumatic spinal cord injury.
